# Extraesophageal Reflux: What Is the Best Parameter for pH-Monitoring Data Analysis from the Perspective of Patient Response to Proton Pump Inhibitors?

**DOI:** 10.1155/2013/736486

**Published:** 2013-01-17

**Authors:** Karol Zelenik, Petr Matousek, Miroslav Tedla, Jakub Syrovatka, Pavel Kominek

**Affiliations:** ^1^Department of Otolaryngology, University Hospital Ostrava, 17 Listopadu 1790, 708 52 Ostrava, Czech Republic; ^2^Faculty of Medicine, University of Ostrava, Syllabova 19, 703 00 Ostrava, Czech Republic; ^3^ENT University Department, Medical School, Comenius University, Bratislava, Antolska 11, 85107 Bratislava, Slovakia; ^4^University Hospital of Coventry and Warwickshire NHS Trust, Clifford Bridge Road, Coventry CV2 2DX, UK

## Abstract

*Objectives*. To analyze the pH-monitoring records of patients with suspected extraesophageal reflux (EER) using three different parameters (number of refluxes (NOR), acid exposure time (AET), and reflux area index (RAI)), with a view to determining which type of analysis is best at selecting the patients who will respond to a proton pump inhibitor (PPI). *Methods*. Demographic data were obtained and the level of the complaint was assessed using the Visual Analogue Scale. A dual probe pH-monitoring study was conducted. NOR greater than six, AET more than 0.1%, and RAI higher than 6.3 mpH were taken to be the thresholds for EER. Subsequently the response to a 12-week PPI trial was analyzed. *Results*. A total of 81 patients were analyzed. The percentages of patients with substantial EER based on NOR, AET, and RAI were 36%, 28% and 26%, respectively. Statistically significant, often positive PPI trials were confirmed in the group identified as having substantial EER using all three types of analysis. When using AET and RAI, the significance was more pronounced (*P* = 0.012 and *P* = 0.013, resp.) in comparison with NOR (*P* = 0.033). *Conclusions*. Patients with EER diagnosed using AET or RAI will respond to PPI significantly often.

## 1. Introduction

**Figure 1:**
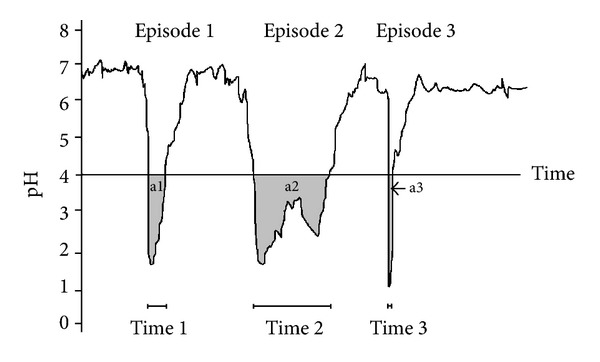
Three different parameters used for pH-monitoring data analysis are compared. There are 3 reflux episodes displayed (Episode 1, Episode 2, and Episode 3). Acid exposure time is the sum of Time 1, Time 2, and Time 3. Reflux area is the sum of calculated a1, a2, and a3 areas.

Ambulatory 24-hour dual probe pH-monitoring remains a widely used diagnostic method for detecting extraesophageal reflux (EER). At present, there is a substantial consensus regarding the methodology for this procedure: the upper probe should be placed above the level of the upper esophageal sphincter (UES) [1]. However, there is still a lack of consensus regarding the interpretation of the data recorded, and physicians continue to argue about what constitutes “normal” and what constitutes pathological EER for most patients. Currently, there are three basic parameters being used for data analysis: number of refluxes (NOR), acid exposure time (AET), and reflux area index (RAI) (Figure 1) [2, 3]. NOR is the sum of all reflux episodes per 24 hours, regardless of their duration and the pH level reached. AET, also sometimes called fraction time, is the percentage of time during the study (usually 24 hours) when the pH is below 4.0. This parameter reflects the severity of EER more objectively. Reflux area (RA) is the sum of the area under the curve for all episodes of pH < 4.0 recorded during the study in units of Ph*minutes. The RAI (in units of mpH) is the RA corrected for the duration of the study. RAI takes into consideration not only the AET but also the level of pH decline and is currently considered the most accurate parameter for measuring the severity of EER (Figure 1) [2, 3].

Every physician who has evaluated recorded pH-monitoring data is familiar with the fact that results may vary with the parameter used for analysis. The question then becomes which parameter is the most precise and best correlates with the response to proton pump inhibitors (PPI). The aim of the present study was to analyze the pH-monitoring records of patients examined for suspected EER using these three different parameters, compare the results, and determine which parameter was the best predictor of a positive response to PPI treatment. To our knowledge, this is the first study to compare all three of these parameters and thereby attempt to establish whether using different criteria has any clinical impact.

## 2. Materials and Methods

The prospective study was performed in accordance with the Declaration of Helsinki, the requirements of good clinical practice, and all applicable regulatory requirements and was approved by the Institutional Review Board. Written informed consent was obtained from all participants before initiating any procedure.

Outpatients aged 18 to 64 years with the complaints commonly attributed to EER (dysphonia, globus, cough, and throat cleaning) lasting more than three months were included in the study, conducted between January 2010 and June 2011. Both those patients who had and those who had not been treated for gastroesophageal reflux disease with a PPI were included in the study, since this fact has no bearing on the aim of the study. Patients with acute upper respiratory infection and oropharyngeal and laryngeal cancer and patients with other serious illnesses (e.g., cardiovascular and neurological complaints, diabetes, and other illnesses) were excluded from the study, because these conditions can significantly aggravate patient complaints. Epidemiologic data (age, sex, BMI, smoking history, bronchial asthma, and professional use of voice) were obtained via questionnaire, and assessment of the severity of the complaints commonly associated with EER (dysphonia, globus, cough, and throat cleaning) was done using the visual analogue scale (VAS). Reflux Finding Scores (RFS) were assessed using rigid video laryngoscopy to determine the level of the laryngeal signs of EER. Afterwards, an ambulatory 24-hour dual probe pH-monitoring study was conducted. A digitrapper pH400 device (Alpine Biomed, Denmark, 2007) with double probes with a fixed distance of 15 cm was used. The proximal sensor was placed immediately above the UES using flexible laryngoscopic guidance (Smit technique). The data recorded were analyzed using GastroTrac software (Alpine Biomed, Denmark, 2007). Upper probe events with pH < 4.0 were only accepted as EER events when Postma'scriteria (the pH decreases to less than 4; the pharyngeal pH drops during or immediately after distal esophageal acid exposure; the pH drop does not occur during an episode of eating; the proximal sensor pH drop is rapid and sharp, not gradual) were met [1]. NOR, AET, and RAI were assessed. NOR greater than six, AET greater than 0.1%, and RAI higher than 6.3 mpH were considered the thresholds for substantial EER [3–6]. Subsequently, all patients were put on a PPI (30 mg lansoprazole) twice a day for twelve weeks and were assessed using VAS at the end of this period to ascertain whether their symptoms (dysphonia, globus, cough, and throat cleaning) had completely vanished, been relieved, or persisted. A drop of at least 3 points in the 10-point VAS, as compared to the VAS value assessed before the PPI trial, was taken to indicate a relief of symptoms, while a decrease of two or less was taken to indicate the persistence of symptoms. A two-sample Student's* t*-test and Fischer's exact test were used to assess differences in RFS and responses to the PPI trial between the groups analyzed. Stata software (version 10) was used for all statistical calculations.

## 3. Results

A total of 90 patients were recruited for the study, nine of whom were excluded from the statistical analysis (five did not tolerate a catheter and four did not come to the last session). A total of 81 patients (31 men and 50 women, mean age 50, SD ± 14) were analyzed (Table 1).

The percentages of patients with substantial EER based on NOR, AET, and RAI were 36% (29 patients), 28% (23 patients), and 26% (21 patients), respectively (Table 2).

Statistically significant higher RFS was confirmed in the group with substantial EER in comparison to the group determined not to have EER using all three types (NOR, AET, and RAI) of analysis (*P* = 0.0166, *P* = 0.0071, and *P* = 0.0007, resp.) (Table 3).

Statistically significant, often positive PPI trials in the group with substantial EER in comparison to the group without EER as determined using all types of analysis (NOR, AET, and RAI) was confirmed as well (*P* = 0.033, *P* = 0.012, and *P* = 0.013, resp.) (Table 4). 

## 4. Discussion

Diagnosing EER and establishing its involvement in patient problems continue to be a challenging and controversial business. This has to do with the complicated pathophysiology of EER and the fact that EER symptoms are nonspecific and vary over time, and moreover with the fact that different patients evince different sensitivities to reflux [1]. The lack of diagnostic criteria for EER and inconsistency in the response to therapy is a source of frustration to many physicians. There is as yet no clear answer to that most important question: “which patients will respond to treatment?” Nevertheless, EER causes very real problems and affects hundreds of thousands of patients annually. It is estimated that up to 10%–15% of all visits to otolaryngology offices are prompted by manifestations of EER [7].

Ambulatory 24-hour dual probe pH monitoring for the detection of EER was introduced by Wiener et al. in 1989 [8]. The methodology involved was refined over the years, and over the last two decades the technique has come to be widely used for the diagnosis of EER. At present, there is a substantial consensus regarding the methodology for this procedure: the upper probe should be placed above the level of the UES. This can be achieved using direct laryngoscopy guidance (Smit technique), or else the position of the UES can be ascertained using manometry [1–3, 9].

The role of pH monitoring in the examination of patients with suspected EER continues to be a contentious issue. Authors who argue that pH testing should be preceded by a PPI trial make a point of stressing inconsistencies in interpretation criteria and unreliability in predicting the response to therapy [9]. On the opposing side, authors who advocate pH testing before a PPI trial point out the risk of PPI overuse: its adverse effects (hip fractures, enteritis, and anaphylactic reaction, among others), the rebound phenomenon when medication is stopped, and the economic impact [10, 11]. Moreover, meta-analysis involving over 790 extraesophageal pH reports in 16 studies over a period of 12 years confirmed that the aggregate number of reflux episodes and the percentage of AET were both significantly greater in persons with EER than in controls [12]. Thus, hypopharyngeal pH-monitoring does appear to be capable of distinguishing persons with EER from normal controls [11, 12].

The dispute over whether pH monitoring or a PPI trial should be used as a first intervention in patients with suspected EER is fuelled by differences in the definitions of physiological and pathological EER adopted by different authors. Some authors consider any pharyngeal reflux abnormal, while others report small amounts of pharyngeal reflux in healthy individuals and consider a small number of EER refluxes (most often three to six refluxes) a threshold for pathological EER [1, 4–6]. Moreover, NOR does not seem to be the best parameter for analysis, because the length and severity of individual EER episodes vary significantly. As a result, two other parameters are currently being used for the analysis of pH monitoring records: AET and RAI. RAI is currently considered the most accurate parameter as it takes into account the severity of the reflux episode, not just its duration (Figure 1) [3].

In the present study, the data recorded during pH-monitoring were analyzed using all three criteria (NOR, AET and RAI). We did not find any studies in the world literature that compared all three criteria. Our results indicate that AET, and RAI are similar parameters and that pathological EER is diagnosed in 28% and 26% of patients, respectively, using these methods. They are more specific and less sensitive in comparison to NOR. Using NOR, pathological EER was diagnosed in 36% of patients. The response to the PPI was significantly higher in patients diagnosed with pathological EER using all three types of analysis. However, when AET and RAI were used, the significance was more pronounced (*P* = 0.012 and *P* = 0.013, resp.) than when NOR was used (*P* = 0.033). In practice this means that if we use more specific types of analysis (AET or RAI) we will diagnose fewer patients with pathological EER, but a higher proportion of diagnosed patients will respond to PPI treatment. This result supports the assertion that the response to a PPI can be predicted by the result of pH testing and that the stricter the criteria adopted for pathological EER, the greater the number of patients responding to PPI treatment.

Similar conclusions can be reached by examining the details of the study published by Hartman [13]. He analyzed five randomized placebo controlled trials which tracked the response to a PPI in patients with suspected EER [13]. In two of them, the effect of the PPI was significantly higher as compared to the placebo, and in one the PPI was reported as possibly having an effect [14–16]. In two other studies, the effect of PPI as compared to the placebo was not confirmed [17, 18]. When we look at these studies closely, a very important fact emerges. In all studies which showed a significant effect of PPI in comparison to the placebo, the diagnosis of EER was arrived at by pH-monitoring, and patients were assigned to the EER group accordingly [14–16]. And conversely, in studies which did not show a significant effect of PPI as compared to the placebo, patients were assigned to the EER group only according to their symptoms and/or signs [17, 18]. Therefore, it can be assumed that, in studies which assigned patients to EER groups without pH testing, more patients are believed to have EER suffered from non-EER laryngitis. This also explains why the effect of PPI in the EER group as compared with the non-EER group did not differ in these studies.

The same result was arrived at in our previous study of patients with globus pharyngeus. In the group of patients with globus pharyngeus and pathological EER as confirmed by pH monitoring, the response to the PPI was significantly higher than in the group of patients with globus pharyngeus but without EER [19].

Even if the use of more specific criteria for the diagnosis of EER improves the practical outcome of pH monitoring, one has to be aware of the limits of this technique [11]. Hence RFS designed by Belafsky is recommended as an important part of the examination of patients with suspected EER, to be used as an adjunct to pH testing [11]. RFS has displayed excellent inter- and intrarater reproducibility [20]. But RFS alone is also limited in specificity because inflammatory changes of the larynx can have many other causes (tobacco, environmental pollutants, infection, excessive voice use, and allergy). Thus, laryngoscopy alone cannot be relied upon to make a diagnosis of EER either, and the combination of laryngoscopy and dual-probe pH testing seems to be of much higher diagnostic sensitivity and specificity for EER [11]. Oelschlager et al. reported that 88% of persons with an abnormal RFS and an abnormal pharyngeal pH test improved with antireflux therapy, as compared with just 44% of persons with an abnormal pH test but normal RFS [21]. This result strongly indicates that the combination of both diagnostic tools offers the best opportunity to accurately secure the diagnosis of EER and reliably predict the response to antireflux therapy.

An additional result of our study was that the sound diagnostic value of RFS was confirmed. RFS was significantly higher in groups of patients with pathological EER diagnosed using all three types of analysis. Moreover, using AET and RAI, which were confirmed to be more specific criteria for the diagnosis of EER, the significance was more pronounced (*P* = 0.0071 and *P* = 0.0007, resp.) in comparison with NOR (*P* = 0.0166).

New devices for the detection of EER—Multichannel Intraluminal Impedance (MII) testing and oropharyngeal pH testing using a Restech device—have emerged recently. The main advantage of MII testing is the ability to detect weakly acidic and alkaline EER episodes. Over the past few years, the device has been used primarily for the examination of impedance below the UES. Normative data for pharyngeal probes have only recently been supplied, by Hoppo [22]. The authors conclude that EER episodes are very rare in asymptomatic populations [22]. The Restech device for the examination of oropharyngeal pH is very sensitive and the examination is well tolerated by patients. Normative data have been available from several recent studies [23–26]. It is very important to keep in mind that even if these new devices seem to be better in terms of their sensitivity to EER, they will raise exactly the same questions as dual probe pH testing has over the past two decades. Most of these have been discussed and summarized in this paper, along with some new perspectives afforded by the results of our study. The most important objective of all methods devised to measure oro- and hypopharyngeal pH is to verify given normative data for different groups of patients and to determine if the results of these tests can predict the response to antireflux therapy. 

## 5. Conclusions

When using AET and RAI in the diagnosis of EER, the significance was more pronounced in comparison with NOR. Using these types of analysis (AET or RAI) we will be able to identify the patients who will respond to PPI treatment.

## Figures and Tables

**Table 1:** Characteristics of the study group.

Patients	*n* = 81
Mean age	50 years (SD ± 14)
Sex	31 male, 50 female
BMI	27,29 (SD = 5.33)
Smokers	*n* = 14 (17.3%)
Voice professionals	*n* = 21 (25.9%)
Bronchial asthma	*n* = 15 (18.5%)

**Table 2:** Number (*N*) and percentage (%) of patients diagnosed with EER (EER^+^) and without EER (EER^−^) using three different parameters of pH monitoring analysis (NOR: number of refluxes, AET: acid exposure time, and RAI: reflux area index).

Parameter	EER^+^ (*N*)	EER^+^ (%)	EER^−^ (*N*)	EER^−^ (%)
NOR	29	35,80	52	64,20
AET	23	28,40	58	71,60
RAI	21	24,93	60	75,07

**Table 3:** Average reflux finding score (RFS) and its standard deviation (SD) in group of patients with extraesophageal reflux confirmed by pH monitoring (EER^+^) and group of patients without EER (EER^−^) using three different parameters (NOR: number of refluxes, AET: acid exposure time, and RAI: reflux area index). The two-sample Student's *t*-test was for statistical analysis of differences between the EER^+^ and the EER^−^ group.

Parameter	EER^+^	EER^−^	*P *
NOR	8.00 ± 3.10	6.31 ± 2.67	0.0166
AET	7.93 ± 2.65	6.15 ± 2.83	0.0071
RAI	8.57 ± 3.00	6.16 ± 2.59	0.0007

**Table 4:** Number of patients with a positive therapeutic trial (TT^+^) and a negative therapeutic trial (TT^−^) in group of patients with extraesophageal reflux confirmed by pH-monitoring (EER^+^) and without EER (EER^−^) using three different parameters (NOR: number of refluxes, AET: acid exposure time, RAI: reflux area index). Fischer's exact test was used for statistical analysis of differences between the EER^+^ and the EER^−^ group.

Parameter	EER^+^		EER^−^		*P *
	TT^+^	TT^−^	TT^+^	TT^−^
NOR	25	4	30	22	0.033
AET	20	3	35	23	0.012
RAI	19	2	36	24	0.013
